# The Galaxy for March Is a Splendid Number

**Published:** 1874-02

**Authors:** 


The Galaxy for March is a Splendid Number.—The leading article is a sketch of Tom Marshall, the great Kentucky Orator and wayward genius. Richard Grant White, the author of "Words and their uses," examines the recently published autobiography of John Stuart Mill. His articles are always instructive and form an admirable feature of the Galaxy. Mr. Justice McCarthy continues his "Linley Rockford," a novel of great beauty and power, as well as furnishing a most interesting sketch of the distinguished French Artist, Gastare Dore. Mrs. Thompson gives a biographical sketch of Johann Sebastian Beck. The ex-Confederate Congressman, Hon. J. L. M. Curry, who since the war has become an eminent Baptist minister, contributes an article upon the Constitution of the Confederate States.

Then come stories, sketches, scientific notes, etc., when the number is closed by the delightful editorial beauties of "Nebulæ," which leaves the reader in an excellent humor and anxious for the arrival of the next number of the Galaxy. Sheldon & Co., 677 Broadway, New York. Price $4.00.

